# *Cryptosporidium* infection in bovine calves: prevalence and potential risk factors in northwest Ethiopia

**DOI:** 10.1186/s13104-018-3219-7

**Published:** 2018-02-07

**Authors:** Abrham Ayele, Zewdu Seyoum, Samson Leta

**Affiliations:** 1 College of Veterinary Medicine and Animal Sciences, University of Gondar, P.O. Box: 196, Gondar, Ethiopia; 2 College of Veterinary Medicine and Agriculture, Addis Ababa University, P.O. Box: 38, Debre-Zeit, Ethiopia

**Keywords:** *Cryptosporidium*, Calves, Prevalence, Risk factors, Northwest Ethiopia

## Abstract

**Objective:**

*Cryptosporidium* is an enteric protozoan organism that causes gastrointestinal disorders in different animals, mainly in calves. The parasite has also a zoonotic importance of children and immunocompromised patients. However, data are limited to northwest Ethiopia. Therefore, we conducted a cross-sectional survey from October 2014 to April 2015 to estimate the prevalence of *Cryptosporidium* infection and to identify potential risk factors in bovine calves in northwest Ethiopia.

**Results:**

Out of the 360 examined calves, *Cryptosporidium* oocysts were recorded in 67 (18.6%) calves. Risk factors such as age, hygiene, faecal consistency, feed source, water source and contact with other domestic animals were significantly (*P* < 0.05) affected the occurrence of *Cryptosporidium* infection. However, significant variations (*P* > 0.05) were not recorded between *Cryptosporidium* infection and gender, body condition score, breed and study sites. Using multivariable analysis, age, feed source, water source, hygiene and close contact with other domestic animals were recognized as potential risk factors for the occurrence of *Cryptosporidium* infection. This study clearly figures out that *Cryptosporidium* infection is prevalent in the study area. Therefore, further studies, extension services and community education are recommended to adopt an integrated control approaches.

## Introduction

*Cryptosporidium* is an intracellular and extra-cytoplasmic protozoan parasite causing gastrointestinal disorders resulting in diarrhoea in young animals and immunocompromised human beings. In cattle, it causes acute or chronic gastrointestinal disturbance, which results in mortality, loss of weight and reduced milk production [1, 2]. The pathogen has a direct life cycle and can develop and multiply in the gastrointestinal epithelial cells of infected animals [3, 4].

To date, about seven species and two genotypes of *Cryptosporidium* have been identified in cattle [5]. The parasite can be transmitted from human to human or animal to animal (anthroponotic transmission) or from animal to human (zoonotic transmission) [6].

Infections are commonly transmitted via the faecal-oral route, sticking direct or collateral contact with infective stages of the fully sporulated oocysts when excreted [7]. The infection is found to be self-limiting in immunocompetent hosts, but it may lead life-threatening acute and chronic diarrhoea in young and immunocompromised animals [8]. The occurrence of cryptosporidiosis in the calf can be determined by age, bed depth and environmental sanitation [1].

Bovine cryptosporidiosis is often misdiagnosed despite the fact that it has been considered as an important cause of neonatal diarrhoea and economic losses in dairy farms [9]. It is characterized by anorexia and diarrhoea, which may result in poor growth rate and death. Clinically, the severity of cryptosporidiosis may be attributed to animals’ age, immune and nutritional status [10, 11].

Various reports have indicated that the prevalence of cryptosporidiosis ranges from 6.25 to 39.65% in cattle in different parts of the world [12–14]. There are various factors that affect the prevalence of cryptosporidiosis including age, bedding type, hygiene, colostrum feeding, management practices, feed and water sources, diarrhoea and climate [15]. In Ethiopia, where over 50 million cattle are raised under various agro-ecological zones, a few research works have been done on bovine cryptosporidiosis in central parts of the country reporting a prevalence ranges from 2.3 to 27.8% [16–18]. However, apart from conducive climatic factors for parasite pathogens, there are limited data on the prevalence and potential risk factors of *Cryptosporidium* infection in calves in northwest Ethiopia. Data regarding the prevalence and potential risk factors of *Cryptosporidium* infection in different agro-climatic zones is crucial to understand the dynamics of transmission, design and establish effective control measures in developing countries, like Ethiopia. Thus, the present study was aimed at estimating the prevalence and potential risk factors of *Cryptosporidium* infection in bovine calves in northwest Ethiopia.

## Main text

### Methods

#### Study area

This present study was conducted in northwest Ethiopia, about 727 km from Addis Ababa, the capital city of the country. The study area is located at a latitude of 35°7′N and longitude of 13°8′E. It also lies at an altitude of 2200 meters above sea level. The annual average temperature and rainfall are about 19.7 °C and 1172 mm, respectively. According to the Central Statistical Agency (CSA) of Ethiopia, the livestock population found in the region include cattle 3.5 mil, shoats 2.5 mil, poultry 2 mil and Equine 400,000. A mixed farming system is the predominant farming practice in the area [19].

#### Study design, study animal and sample size

A cross-sectional study design with a simple random sampling procedure was employed from October 2014 to April 2015 to address the above-mentioned objectives. The study animals were both Holstein-cross and local breed calves aged up to 12 months. The sample size was determined by the formula which has been given by Thrusfield [20] using an expected prevalence of 17.6% [16] at 95% confidence interval and 5% precision value. Therefore, a sample size of 223 calves was obtained. However, a total of 360 dairy calves were recruited to collect the required faecal samples.

#### Sample collection and laboratory analysis

Fresh faecal samples were collected directly from the rectum of a calf and kept in a separate clean bottle in a cold box. At the time of sample collection, the name of the owner, sampling date, faecal consistency (normal/diarrhoea), presence/absence of close contact with other domestic animals, feed and drinking water source, hygiene status and the calf age, sex, breed and body condition score were recorded for each animal on a recording sheet. After collection, the samples were then transported to the laboratory on the same day of collection for further process. The samples were then processed using faecal flotation with a Sheather’s sugar solution [21]. Faecal smears were also prepared and stained using modified Ziehl–Neelsen acid-fast stain [22].

#### Data analysis

The raw data were managed and summarized using STATA version 11. Descriptive statistics were utilized to summarize the raw data. The percentage of *Cryptosporidium* infection was calculated by dividing the number of infected animals by the total number of animals examined multiplied by 100. Univariate logistic regression method was used to determine the association between potential risk factors and occurrence of *Cryptosporidium* infection. Variables with significance at *P* < 0.05 were selected for further multivariate logistic regression. Multicollinearity test was also made to check the multivariate model fit and to identify confounding factor. The results were considered statistically significant when the *P* value is ≤ 0.05.

### Results

Out of the 360 examined calves, *Cryptosporidium* oocysts were detected in 67 (18.6%) calves. By using univariate logistic regression analysis, six risk factors were identified that affect the prevalence of *Cryptosporidium* infection in calves (Tables 1 and 2). Calf age significantly affected the prevalence (OR 2.12, 95% CI 1.12–3.91, *P* < 0.05); calves under 6 months old were highly affected (28.4%) than older calves. Feed source was also found to affect the occurrence of *Cryptosporidium* infection (OR 2.26, 95% CI 1.22–4.33, *P* < 0.01). Feeding on milk and pasture resulted in a higher infection rate (23.4%) than pasture alone. Similarly, water source significantly affected the infection rate (25.13%); getting river water was found to expose calf three times more for *Cryptosporidium* infection than tap water. Furthermore, hygiene status also significantly influenced the prevalence of infection (OR 2.92, 95% CI 1.51–5.55, *P* < 0.001). It was three times more in poor hygiene calves (34.4%) than hygienic calves (15.2%).

**Table 1:** Univariable analysis of host-related risk factors with *Cryptosporidium* oocyst shedding by calves

Potential risk factors	No. animals	No +ve	Prev. (95% CI)	OR (95% CI)	*P* value
Breed					
Indigenous	271	48	17.7 (13.6–22.7)	1	
Holstein-cross	89	19	21.3 (14.1–31)	1.26 (0.7–2.29)	0.445
Sex					
Female	198	36	18.2 (13.4–24.1)	1	
Male	162	31	19.1 (13.8–25.9)	1.065 (0.63–1.81)	0.817
Age					
6–12 months	279	44	15.8 (12–20.5)	1	
< 6 months	81	23	28.4 (19.7–39.0)	2.12 (1.2–3.78)	0.011
Body condition scores					
Good	271	50	18.5 (14.3–23.5)	1	
Poor	89	17	19.1 (12.3–28.5)	1.04 (0.57–1.92)	0.89
Overall prevalence	360	67	18.6 (14.9–23)

**Table 2:** Univariable analysis of non-host related risk factors to the prevalence of *Cryptosporidium* infection

Potential risk factors	No. animals	No. +ve animals	Prevalence (95% CI)	OR (95% CI)	*P* value
Study site					
Urban	149	22	14.8 (10–21.4)	1	
Peri-urban	211	45	21.3 (16.3–27.3)	1.57 (0.89–2.74)	0.117
Feed source					
Pasture alone	151	18	11.9 (7.7–18.1)	1	
Milk + pasture	209	49	23.4 (18.2–29.6)	2.26 (1.26–4.07)	0.006
Water source					
Pipe	165	18	10.91 (7.0–16.6)	1	
River	195	49	25.13 (19.6–31.7)	2.74 (1.52–4.93)	0.001
Hygienic status					
Good	296	45	15.2 (11.6–19.7)	1	
Poor	64	22	34.4 (23.9–46.6)	2.92 (1.6–5.35)	0.001
Faecal consistency					
Normal	298	45	15.1 (11.5–19.6)	1	
Diarrhoeic	62	22	35.5 (24.7–47.9)	3.09 (1.68–5.69)	0.001
Contact with domestic animals					
No	64	6	9.4 (4.4–18.9)	1	
Yes	296	61	20.6 (16.4–25.6)	2.51 (1.03–6.09)	0.042
Overall prevalence	360	67	18.6 (14.9–23)		

Analysis of faecal consistency as a risk factor shows a significant variation in the occurrence of *Cryptosporidium* infection in calves with diarrhoea and normal faecal matter (OR 3.09, 95% CI 1.59–5.9, *P* < 0.001). Thus, the occurrence of infection was three times higher in diarrheic calves than those with the normal faecal matter. Mixing of calves and other domestic animals also had a significant effect on the risk of infection. Calves which had free interaction with other domestic animals had a higher prevalence than those calves kept separately (OR 2.51, 95% CI 1.02–7.44, *P* < 0.05). On the contrary, location, gender, body condition score and breed did not show significant (*P* > 0.05) effect on *Cryptosporidium* infection rate.

Multivariable analyses were also done for those significant variables in univariable analysis and the test result showed that age, feed source, water source, hygiene and contact with other domestic animals were significant variables (*P* < 0.05); where calves < 6 months, milk plus pasture consumption, river water, poor hygiene and close contact with other domestic animals showed significant effect on the occurrence of *Cryptosporidium* infection in calves (Table 3).

**Table 3:** Multivariable logistic analysis of risk factors that were significant using univariable analysis as shown in Tables 1 and 2

Risk factors	OR	SE	z	P > |z|	95% confidence interval	
Calves < 6 month	2.376	0.763	2.69	0.007	1.266	4.457
Milk plus pasture consumed	2.927	0.947	3.32	0.001	1.553	5.519
River water	2.661	0.859	3.03	0.002	1.414	5.009
Poor hygiene	2.625	0.872	2.90	0.004	1.368	5.035
Contact with domestic animals	2.599	1.241	2.00	0.045	1.019	6.627

### Discussion

In this study, the overall prevalence of *Cryptosporidium* infection in calves was found to be 18.6% (67/360). This is in line with the report of Abebe et al. [16] who noted 17.6% infection rate in dairy calves in Central Ethiopia. Similarly, Lefay et al. [23] from France and Geurden et al. [24] from Zambia reported a comparable prevalence in dairy calves. However, Santin et al. [25] in the USA, Nguyen et al. [26] in Vietnam and Brook et al. [1] in the UK reported relatively higher occurrence in calves. This variation between reports might be ascribed to the geographic difference, study design, diagnostic techniques, production system and management as well as the season of the year when the study was conducted. According to Venu et al. [13], the diagnostic techniques employed in the present survey are less sensitive and can give false negative results. This might also be the reason why report variation recorded.

The effect of age was confirmed in the present study as of Geurden et al. [24], Venu et al. [13] and Joute et al. [14] who noted the significant effect of age on the prevalence of *Cryptosporidium* infection in animals. Similarly, Noordin et al. [27] affirmed the importance of age on the occurrence of *Cryptosporidium* infection. This supports the present finding in which higher prevalence recorded in calves older than 6 months. This could be owing to the fact that the immature immune system of young calves. Brook et al. [1] also reported calves under 4 months of age are more at risk for *Cryptosporidium* infection. This is also supported by Kvac et al. [28] who described that resistance to infection could be developed with age due to immune development through time.

A strong association was also observed between the hygienic status of calves and their house, and the occurrence of *Cryptosporidium* infection in calves. The present report is supported by the finding of Abebe et al. [16] who described the substantial connection of the *Cryptosporidium* infection with the hygienic status of dairy animals and their farms. Similarly, El-Khodery and Osman [21], and Castro-Hermida et al. [29] affirmed that poor hygiene increases the infection rate and spread *Cryptosporidium* species in animals. This might be ascribed to that dirty and muddy farm could presumably build a favourable microclimatic factors or conditions for the presence or survival of *Cryptosporidium* oocysts in the farms or animal houses. This increases feed and water contamination, which in turn might favour the exposure of calves to *Cryptosporidium* infection.

A significant association was also observed between the prevalence of *Cryptosporidium* oocysts and faecal consistency of calves; where diarrhoeic animals had shed the oocysts more frequently than those calves with the normal faecal matter. This is in accordance with El-Khodery and Osman [21], Causape et al. [30] and Lise et al. [31] who reported a strong association between *Cryptosporidium* oocyst shedding and calf diarrhoea. Thus, it seems that *Cryptosporidium* is the enteropathogen which strongly associated with diarrhoea. This might be due to the fact that the pathogen causes villous atrophy and crypt hyperplasia, which results in a decrease in the absorptive surface area of the intestine; thus glucose, water and sodium absorption are hindered and results in diarrhoea [7]. Moreover, the parasite could have a capability in reducing disaccharidase activity resulting in the reduced breakdown of sugars resulting in bacterial overgrowth, the formation of volatile fatty acids, and changes in osmotic pressure; these changes, then cause the characteristic severe and watery diarrhoea [7, 32].

In our study, having river water also significantly affected the occurrence of *Cryptosporidium* infection in calves. This is supported by the report of El-Khodery and Osman [21] who accounted that water supply can affect the prevalence of cryptosporidiosis. This might be attributed to the contamination of wells/rivers to drain or runoff from farming areas [33].

*Cryptosporidium* species can affect different animals, including human beings [16]. The parasite can also be transmitted directly or indirectly through faecal-oral route from the infected animal to healthy animals [6, 23]. Therefore, mixing of different species of animals may help in contraction and spread of the infection [22]. This supports the present report in which it shows that the significant effect of direct contact between calves and other domestic animals on *Cryptosporidium* infection. Similarly, Mohammed et al. [34] reported the tendency of infection risk can be reduced when animals kept individually or without close contact with different species of animals.

In conclusion, this study clearly figures out that *Cryptosporidium* infection is prevalent in northwest Ethiopia. The prevalence of *Cryptosporidium* infection in calves was found to be 18.6%. Therefore, awareness creation should be practised in the community about public health and economic significances of cryptosporidiosis and about the proper care to be involved. In addition, further epidemiological studies involving different risk factors need to be conducted.

## Limitations

In this study, we recognized the following limitations:

- The study was a cross-sectional study, which could not determine the causal effect relationships between independent and dependent factors.
- The study did not involve species identification, molecular characterization and seasonal investigation, which may limit the value of the report.

## References

[1] Brook E, Hart CA, French N, Christley R (2008). Prevalence and risk factor of *Cryptosporidium* infection in young calves. Vet Parasitol.

[2] Smith HV, Caccio SM, Tait A, McLauchlin J, Thompson RC (2006). Tools for investigating the environmental transmission of *Cryptosporidium* and *Giardia* infection in humans. Trends Parasitol.

[3] Chen W, Harp JA, Harmsen AG (2003). *Cryptosporidium parvum* infection in gene-targeted B cell-deficient mice. J Parasitol.

[4] Shafieyan H, Alborzi A, Hamidinejat H, Tabandeh MR, Hajikolaei MRH (2014). Prevalence of *Cryptosporidium* species in ruminants of Lorestan province, Iran. J Parasit Dis.

[5] Hunter PR, Hughes S, Woodhouse S, Syed Q, Verlander NQ, Chalmers RM, Morgan K (2005). Sporadic cryptosporidiosis case–control study with genotyping. Emerg Infect Dis.

[6] Abu-Madi MA, Behnke JM, Ismail A, Al-Olaqi N, Al-Zaher K, El-Ibrahim R (2012). Comparison of intestinal parasitic infection in newly arrived and resident workers in Qatar. J Parasit Vectors.

[7] Radostits OM, Gay CC, Hinchclif KW, Constable PD (2007). Veterinary medicine: a textbook of the disease of cattle, horse, sheep, pig and goats 10th edition Spain.

[8] Snelling WJ, Xiao L, Ortega-Pierres G, Lowery CJ, Moore JE, Rao JR, Smyth S, Millar BC, Rooney PJ, Matsuda M, Kenny F, Xu J, Dooley JS (2007). Cryptosporidiosis in developing countries. J Infect Dev C.

[9] Goma FY, Geurden T, Siwila J, Phiri IGK, Gabriel S, Claerebout E, Vercruysse J (2007). The prevalence and molecular characterization of *Cryptosporidium* species in small ruminants in Zambia. Small Rumin Res.

[10] Urquhart GM, Armour J, Duncan JL, Dunn AM, Jennings FW (1996). Veterinary parasitology.

[11] Nasir A, Avars M, Khan MS, Ahmed N (2009). Prevalence of *Cryptosporidium parvu*m infection in Lahore (Pakistan) and its association with diarrhoea in dairy calves. Int J Agric Biol.

[12] Azami M (2007). Prevalence of *Cryptosporidium* infection in cattle in Isfah, Iran. J Eukaryot Microbiol.

[13] Venu R, Latha BR, Bath AS, Sreekumar C, Raj GD, Raman M (2013). Factors influencing on the prevalence of *Cryptosporidium* infection in South Indian dairy calves. J Parasit Dis.

[14] Joute JR, Gill JPS, Singh BB (2014). Prevalence and molecular epidemiology of *Cryptosporidium parvum* in dairy calves in Punjab (India). J Parasit Dis.

[15] Ogendo A, Obonyo M, Wasswa P, Bitek A, Mbungua A, Thumbi SM (2017). *Cryptosporidium* infection in calves and the environment in Asembo, Western Kenya. Pan Afr Med J.

[16] Abebe R, Wossene A, Kumsa B (2008). An epidemiological study of *Cryptosporidium* infection in dairy calves on selected dairy farms of central Ethiopia. Revue Méd Vét.

[17] Regassa A, Gizaw O, Abunna F, Abebe R, Beyene D, Megersa B, Debela E, Asmare K, Skierve E (2013). *Cryptosporidium* in calves, lambs and kids in Haramaya, eastern Ethiopia. Ethiop Vet J.

[18] Teklu W, Adamu H, Petros B (2013). Prevalence of *Giardia duodenalis* and *Cryptosporidium* species infections among children and cattle in North Shewa Zone, Ethiopia. BMC Infect Dis.

[19] Central Statistical Agency (CSA) of Ethiopia Annual Report of 2014; 2015.

[20] Thrusfield M (2005). Veterinary epidemiology.

[21] El-Khodery SA, Osman SA (2008). Cryptosporidiosis in buffalo calves (*Bubalus bubalis*): prevalence and potential risk factors. Trop Anim Health Prod.

[22] Maurya PS, Rakesh RL, Pradeep B, Kumar S, Kundu K, Garg R, Ram H, Kumar A, Banerjee PS (2013). Prevalence and risk factors associated with *Cryptosporidium* species infection in young domestic livestock in India. Trop Anim Health Prod.

[23] Lefay D, Naciri M, Vitovec J (2006). Prevalence of *Cryptosporidium* infection in calves in France. Vet Parasitol.

[24] Geurden T, Gomma FY, Siwilw J, Phri IGK, Mwanza AM, Gabrie LS, Claerboute VJ (2006). Prevalence and genotyping of *Cryptosporidium* in three cattle husbandry systems in Zambia. Vet Parasitol.

[25] Santin M, Trou TJM, Xiao L, Zhou L, Greiner E, Fayer R (2004). Prevalence and age-related variation of *Cryptosporidium* species and genotypes in dairy calves. Vet Parasitol.

[26] Nguyen ST, Nguyen DT, Quyet LD, Le Hua IN, Nguyen TV, Honma H, Nakai Y (2007). Prevalence and first genetic identification of *Cryptosporidium* species in cattle in central Vietnam. Vet Parasitol.

[27] Noordin F, Rajapakse RPVJ, Faizal ACM, Horadagoda NU, Arulkanthan A (2000). Prevalence of *Cryptosporidium* infection in goats in selected locations in three agroclimatic zones of Srl-Lanka. Vet Parasitol.

[28] Kvac M, Kouba M, Vitovec J (2006). Age-related and housing dependence of *Cryptosporidium* infection of calves from dairy and beef herds in South Bohemia Czech Republic. Vet Parasitol.

[29] Castro-Hermida JA, Gonzalez-Losada YA, Ares-Mazas E (2002). Prevalence of and risk factors involved in the spread of neonatal bovine cryptosporidiosis in Galicia (NW Spain). Vet Parasitol.

[30] Causape AC, Quilez J, Sanchez-Acedo C, del Cacho E, Lopez-Bernad F (2002). Prevalence and analysis of potential risk factors for *Cryptosporidium parvum* infection in lambs in Zaragoza (Northeastern Spain). Vet Parasitol.

[31] Lise A, Trotz-Williams S, Wayn M, Leslie KE, Duffield T, Nydam DV, Peregrine S (2007). Calf level risk factors for neonatal diarrhoea and shedding of *Cryptosporidium parvum* in Ontario dairy calves. Prev Vet Med.

[32] Rickard LB (2001). Veterinary parasitology: the practical veterinarian. Printed in the United States of America.

[33] Adamska M (2015). Molecular characterization of *Cryptosporidium* and *Giardia* occurring in natural water bodies in Poland. Parasitol Res.

[34] Mohammed HO, Wade SE, Schaaf S (1999). Risk factors associated with *Cryptosporidium parvum* infection in dairy cattle in southeastern New York State. Vet Parasitol.

